# Biodrying of Mixed Food-Waste Fractions Containing Packaging Plastics: Effects on Moisture Content, Calorific Value and Compost Quality

**DOI:** 10.3390/ma19132739

**Published:** 2026-06-26

**Authors:** Jakub Pulka, Mariusz Siudak, Andrzej Lewicki, Wiktor Bojarski, Mateusz Nowak, Mariusz Stanisławczyk, Wojciech Czekała

**Affiliations:** 1 Department of Biosystems Engineering, Poznań University of Life Sciences, Wojska Polskiego 50, 60-627 Poznań, Poland; jakub.pulka@up.poznan.pl (J.P.); andrzej.lewicki@up.poznan.pl (A.L.); wiktor.bojarski@up.poznan.pl (W.B.); mateusz.nowak@up.poznan.pl (M.N.); 2 AG Energy Sp. z o.o., Floriana Piotrowskiego 4/19, 10-692 Olsztyn, Poland; m.siudak@agenergy.pl; 3 Mazbest Sp. z o.o., Plac Bankowy 2, 00-095 Warszawa, Poland; mazbest@interia.pl

**Keywords:** biodrying, food waste, waste valorization, moisture content, unpacked plastics, plastic contaminations, circular economy

## Abstract

Approximately 30% of the food produced worldwide is wasted, and a substantial share of municipal food waste still contains non-biodegradable packaging material after sorting. This study investigated an aerobic biodrying process for reducing the moisture content of mixed food-waste fractions, containing varying proportions of green biomass, vegetables, kitchen waste, and packaging-derived plastics, in order to increase their calorific value and obtain a refuse-derived fuel (RDF). Four substrate variants (K1–K4, including a control without added plastics) were biodried in laboratory-scale bioreactors. Process temperatures exceeded 70 °C in all variants, and the addition of plastics increased both the cumulative and the average temperature relative to the control. The plastic fraction recovered after biodrying showed the largest increase in calorific value, reaching over 15 MJ∙kg^−1^, while the AT4 respiration activity of the separated compost fraction decreased to around 10 mg O_2_ g^−1^ DM in all variants, indicating good storage stability. The results suggest that pre-treated plastics did not adversely affect the biodrying process and, owing to their structuring properties, may support biological decomposition of the remaining biomass; these preliminary, single-run findings should be confirmed in replicated trials.

## 1. Introduction

The widespread use of fossil fuels was first driven by the Industrial Revolution [1]. The supply of such energy sources, considered limitless at that time, has nowadays become almost depleted [2,3]. The dependence of the global economy on fossil fuels has contributed to this depletion [4,5]. The energy crisis, which the world now faces, is the effect of this shortage [2]. Several factors have led to it, including the constantly increasing energy demand, insufficient reliance on renewable energy, and low public awareness of the importance of energy saving [6]. This pre-existing, structural energy crisis was further aggravated by more recent disruptions, such as the COVID-19 pandemic and the war in Ukraine, which affected the global fuel market and the balance of energy supply and demand. As a result of these aggravating factors, the patterns of international trade have changed, and the fluctuations of petroleum prices have increased [7]. All of these events have only deepened the global energy crisis. Most recently, escalating geopolitical tensions in the Middle East have led to disruptions of shipping through the Strait of Hormuz, a critical chokepoint for global oil and gas trade, triggering sharp spikes in crude oil prices and further illustrating the volatility and supply risk inherent to fossil-fuel-based energy systems [8].

The exploitation of fossil fuels has also resulted in the accumulation of carbon dioxide and other greenhouse gases in the atmosphere [9,10]. According to the literature, power plants operating on fossil energy sources emit 33.4 Gt of carbon dioxide each year which constitutes 40% of the total annual emission of this gas [11]. This justifies addressing the energy and climate crises both at the same time [5].

The demand for electric energy is prognosed to increase by 50% by 2030 relative to the 2020 level [12]. This will impact the demand for energy from renewable resources [13,14,15]. The inconsistent rate of generation of energy from sources such as wind or sunlight, however, demands developments in energy transport and storage. The production of energy from biomass is less affected by these problems [11,16]. Biomass may be used as feed in biogas installations [17,18].

The utilization of biogas, a biofuel, results in reduced greenhouse gas emissions compared to fossil fuels. Moreover, the technology of its production allows the use of various feedstocks [18,19]. The raw materials used in biogas plants include agricultural by-products, communal and industrial biowaste, and waste from animal production—manure and slurry [20,21,22]. While many European biogas plants are operated with corn silage and manure, food waste has attracted interest as an alternative feedstock. There are not many installations in Europe which function using this waste. At the same time, the substantial amount of food waste generated in the region creates an attractive opportunity, as it is estimated at 80 Mt [20,23]. This type of feedstock is characterized by a high moisture content which limits its suitability for incineration or pyrolysis. Such applications would demand its initial drying—an energy-consuming process [20]. This further adds to the attractiveness of food waste as a biogas feedstock which has additionally been reported to show good biogas yield potential [24,25,26].

Food waste is generated by retailers and consumers. It should be noted, however, that not all of the biodegradable waste generated by communities falls into this category. Specific food waste examples include waste such as expired food products or food products unfit for consumption and biodegradable kitchen waste which were assigned the following codes according to the Polish list of wastes: 16 03 80 and 20 01 08, respectively [27,28]. Of note, this type of waste also contains non-biodegradable matter, for example that originating from packaging. Separation of the non-biodegradable fraction is thus necessary in order to make food waste a suitable substrate for biogas production. Such pretreatment leads to the formation of a new fraction which is mainly composed of packaging materials. These materials could be utilized by combustion.

While packaging materials have a significant energetic potential [29,30,31], the ones separated from food waste problematically contain large amounts of moisture. This is a result of an imperfect purification procedure after which some biodegradable matter remains within the non-biodegradable fraction. This includes juices and other liquids that are especially difficult to remove form the packaging materials. Such a situation negatively impacts the economy of combustion as a large energy input is wasted on water evaporation. In order to make combustion of such wastes effective, the combusted material must be dried first. Among the currently available drying technologies, biodrying seems promising.

Biodrying is an aerobic decomposition process similar to composting [32,33,34]. It is used for removing water from highly moist materials containing organic matter. In this process, the biochemical activity of microorganisms supplies heat that facilitates water evaporation. Convection and molecular diffusion also take part in the elimination of water. The process is performed in completely or partially sealed reactors [35,36,37]. Biodrying results in the preservation of the energetic potential as the removal of organic matter is insignificant. This leads to obtaining high quality solid fuels, such as refuse-derived fuel (RDF) or solid recovered fuel (SRF) [35].

The use of the biodrying process ensures the preservation of the energy potential of the dried waste while consuming minimal organic matter. In view of this, the waste after the discussed process can be high-quality renewable solid fuels (e.g., RDF—refuse-derived fuel or SRF—solid recovered fuel). Refuse-derived fuel (RDF) and solid recovered fFuel (SRF) are types of alternative fuels created from waste. RDF is derived from household and business waste, containing both biodegradable materials and plastics. Non-combustible components like glass and metals are removed, and the remaining materials are shredded. In contrast, SRF is a high-quality, alternative fuel produced primarily from commercial waste, such as paper, cardboard, wood, textiles, and plastics. SRF undergoes additional processing to improve its quality and value. It has a higher calorific value, making it more valuable due to the level of heat it is capable of generating [35,38,39,40].

While biodrying of source-separated organic waste was investigated previously, most existing studies focused either on the dewatering and calorific-value aspects relevant to RDF/SRF production, or on the agronomic quality of the resulting biosolids as a soil improver, but rarely both within a single experimental framework. Moreover, the use of dried packaging plastics recovered during food-waste unpacking as a structuring material for biodrying has not been systematically evaluated. This study addresses this gap by simultaneously assessing the following: (i) the effectiveness of biodrying in reducing moisture and increasing calorific value for alternative-fuel production, and (ii) the suitability of the resulting compost as a soil improver and of the recovered plastics as a structuring agent, thereby linking the waste-to-energy and waste-to-soil-improver pathways within one process.

This paper aims to answer the research problems expressed in the form of these questions. Does the process of biodrying waste effectively remove excess moisture, thereby increasing its calorific value enabling the production of alternative fuel in the form of RDF? Will the resulting biosolid compost meet the standards of a soil improver, and will the dried plastics be a suitable structuring material for the process? Based on the mentioned research problems, the following research hypotheses were developed:

- Removal of excess moisture from the substrate mixtures by means of the biodrying process will contribute to increasing their calorific value making it possible to use these substrates as alternative RDF fuel.
- The quality of the compost obtained after the biodrying process will be comparable to the quality of soil improvers. This hypothesis is evaluated against AT4 respiration-activity thresholds for soil improvers in Section 3.6 and discussed further in Section 3.7.
- The dried plastics will be an effective structuring material in the biodrying process, supporting the biological decomposition of organic matter in the bioreactor.

## 2. Materials and Methods

### 2.1. Substrates

The raw materials used were acquired from a company producing substrates for biogas plants. Four categories of raw materials were used: kitchen waste, vegetables, green biomass with a high content of dry grass, and packaging materials separated from food waste. Table 1 contains the basic parameters of the substrates used in the study.

The biodrying process was performed using four substrates composed of these raw materials in varied proportions and named from K1 to K4. Their composition is presented in Table 2 alongside their basic properties. The compost mixture placed in the bioreactor K3 was the control sample for the conducted experiment. This mixture included only green biomass and vegetables (cabbage/lettuce leaves). These are the materials that are included in any other compost mixture. This arrangement made it possible to compare the effects of additional components of the prepared mixtures on the composting process. The total mass of the input raw material was 22 kg in each of the composting vessels; however, owing to the differences in the composition, the bulk density varied—such a mass of compost mixtures was sufficient due to their low density, making them fill the entire space of the bioreactors used in the study.

The proportions of the individual raw materials in each substrate mixture (K1–K4) were selected based on several criteria: the resulting carbon-to-nitrogen (C/N) ratio, the dry matter content and bulk density of the mixture, and an industrial requirement to incorporate as high a proportion as possible of the target packaging-plastic fraction, reflecting the composition of the residual stream generated by mechanical de-packaging of food waste.

### 2.2. Experimental Setup

The research was conducted in the Ecotechnologies Laboratory of the Poznań University of Life Sciences. Custom designed two-walled isothermal bioreactors were used. The exact scheme of these bioreactors is presented in the referenced articles [41,42]. The suitability of this type of design was confirmed in many previous experiments [43,44,45]. The thermal insulation used allowed the upkeep of proper process conditions. The system was designed to realistically reflect composting in a turned windrow system and to enable precise monitoring of the process. Previous comparative experiments proved that the result obtained using this system complied with ones obtained in real industrial installations. The working volume of the bioreactors was 165 dm^3^. At the start of the experiment the substrates were thoroughly mixed and placed within the bioreactors. Then, the vessels were hermetically seals, and aeration was started.

### 2.3. Analytical Methods

#### 2.3.1. Temperature Measurement

Three layers can be distinguished in compost windrows: surface, core, and bottom—the one closest to the ground. Considering the fact that temperature kinetics may differ significantly in each of these layers, the measurement was conducted throughout the whole height of the chamber with 5 cm gaps using a specialized sensor rod Pt100 WIKA (Włocławek, Poland). The value of the accumulated temperature was calculated by summing the readings obtained with a constant frequency of 1 measurement every 12 h.

#### 2.3.2. pH Measurement

A portion of 20 g of fresh substrate was suspended in 180 mL of water and the measurement of the prepared suspension was performed with an Elmetron CX-401 (Zabrze, Poland).

#### 2.3.3. Determination of Dry Matter, Dry Organic Matter, and Ash

The analyses were performed at the initial and final points of the experiment and at the time of turning. The samples obtained from the windrow were homogenized, weighed, and dried at a temperature of 105 °C for 24 h in Thermo Scientific HeraTherm Oven (Waltham, MA, USA). After the determination of dry matter, the samples were mineralized at 550 °C in a Czylok FCF 7SM muffle furnace (Jastrzębie-Zdrój, Poland) in order to determine the contents of organic matter and ash according to the EN 15935:2021 standard [46].

#### 2.3.4. Determination of Mass and Volume of the Windrows

The windrow volume was determined at the start and finish of the experiment. The mass of the windrows was determined using a laboratory scale Radwag MYA 2.4Y PLUS balance (Radom, Poland) with an accuracy of 0.1 kg.

#### 2.3.5. Gas Emission Analysis

Gases are released from the bioreactor during composting. Oxygen and carbon dioxide are the most important of them, as their ratio indicates whether the process proceeds properly or not. The qualitative and quantitative analysis of the off-gas was performed with a GA5000 gas analyzer (Geotech GA5000, Dexter, MI, USA). The device is certified according to the requirements of ATEX II 2G Ex ib IIA T1 Gb (Ta = −10 °C to +50 °C), IECEx, CSA and ISO/IEC 17025:2017 [47]. It allows the monitoring of the following gases (measurement ranges in parentheses): O_2_ (0–25%), CO_2_ (0–100%), CH_4_ (0–100%), NH_3_ (0–1000 ppm), and H_2_S (0–10 000 ppm). The instrument was calibrated weekly using calibration gases: a mixture of 65% CH_4_ and 35% CO_2_, 2500 ppm H_2_S and 250 ppm NH_3_ in nitrogen. The oxygen sensor was calibrated with air.

#### 2.3.6. Ammonium Nitrogen in Liquid Streams

During the process, leachates and condensates are formed. These were collected, weighed and analyzed for the content of ammonium nitrogen. A total of three liquid streams were analyzed: leachate from the vessel, condensate from the vessel, and condensate from the exhaust cooler.

#### 2.3.7. Calorific Value

Samples were dried at 105 °C, ground, and analyzed for higher heating value with an IKA C200 calorimeter (Staufen, Germany) with Sartorius QUINTIX 125D-1CEU balance (Göttingen, Germany) in accordance with ISO 18125:2017 [48], using Equation (1).

(1) $$Q_{s}^{a}=\frac{C\left(D_{t}-k\right)-c}{m}\left[\frac{\mathrm{kJ}}{\mathrm{kg}}\right]$$

*C*—heat capacity of the calorimeter [J/°C]

*D_t_*—overall temperature rise of the main period [°C]

*k*—ambient heat transfer correction [°C]

*c*—sum of corrections for additional thermal effects [J]

*m*—sample weight [g]

(2) $$Q_{i}^{a}=Q_{s}^{a}-24.42\left(W^{a}-8.94H^{a}\right)\left[\frac{\mathrm{kJ}}{\mathrm{kg}}\right]$$

$Q_{s}^{a}$—average heat of combustion value of the fuel [J/g]

24.42—heat of vaporization of water at 25° corresponding to 1% water in the fuel [J/g]

*W^a^*—moisture content of the analytical fuel sample [%]

8.94—hydrogen-to-water conversion factor

*H^a^*—hydrogen content of the test sample

Percentage content of the selected elements. A GA 2000 elemental analyzer (Thermo Scientific FlashSmart elemental analyzer (Waltham, MA, USA)) with Sartorius QUINTIX 125D-1CEU balance (Göttingen, Germany) was used to determine the percentage of the elements C, H, N, S, and O. This analysis was carried out on a previously dried and ground sample.

Respiration activity (AT4). Respiratory activity was measured using an OxiTop measuring kit (WTW/Xylem, Weilheim, Germany). For this purpose, a 40–65 g sample of fresh material was placed in a measuring cylinder. The volume of this sample was recorded and placed in a 2500 mL glass reaction bottle. The bottles prepared in this way were kept at room temperature. In a glass beaker at the top of the reaction bottles, soda lime pellets were placed with an indicator. This allowed the carbon dioxide produced to be absorbed, and a negative pressure was created in the system, which was measured using the measuring heads of the OxiTop kit. The value of oxygen consumed was calculated using the following equation.

(3) $${AT}_{4}\left[mgO_{2}/DM\right]=∆p\;*\;\frac{M_{O2}}{R\;*\;T}\;*\;\frac{V_{ges}-V_{abs}-V_{sample}}{m_{sample\;dry}}$$

∆*p*—difference in pressure [hPa],

*M*_*O*2_—the molecular mass O_2_ [=31.998 mg mol^−1^],

*R*—the ideal gas constant [=83.140 mL hPa (K mol)^−1^],

*T*—the temperature in Kelvin [=293.15 K],

*V_ges_*—the total volume [=2500 mL],

*V_abs_*—the volume of medium of absorbance [mL],

*V_sample_*—the volume sample [mL],

*m_sample dry_*—the dry mass of sample [g DM].

## 3. Results

### 3.1. Temperature

**Figure 1:**
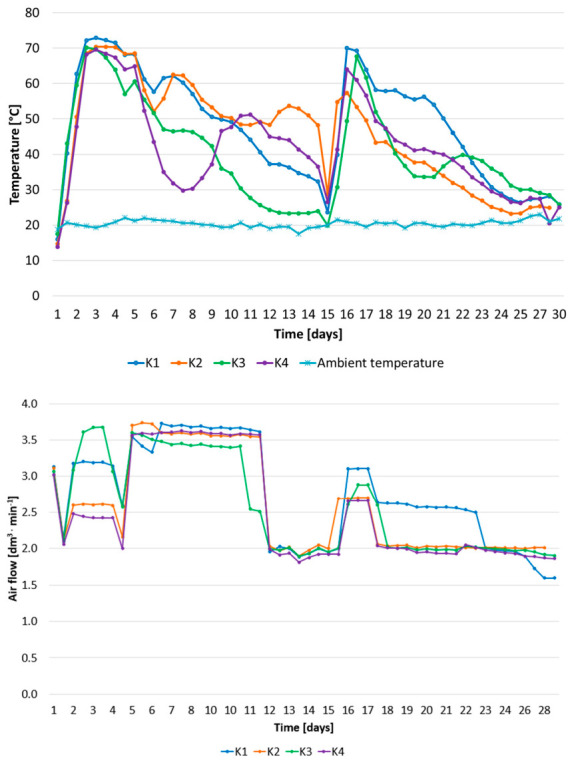
Temperature changes and airflow in the bioreactors.

As already mentioned, biodrying is a variation of composting [36,49]. Thus, similarly to composting, measurement of temperature is essential [50,51,52]. It decides the rate of biochemical reactions and its kinetics are determined by the activity of aerobic microorganisms [53,54]. Figure 1 shows the temperature changes in the bioreactors throughout the course of the biodrying experiment.

In all the bioreactors, the temperature peaked after the first 48 h. The maximum temperature values were 72.94 °C, 70.40 °C, 70.19 °C, 69.58 °C for bioreactors K1, K2, K3, and K4, respectively. Differences in the temperature kinetics were noticed between the runs with K4 showing the quickest rate of temperature decrease, and K3—the slowest.

Accumulated temperature is another parameter that can be analyzed in order to study the differences in heat generation and loss between the tested substrate mixtures. The values of accumulated temperature were 2545 °C, 2366 °C, 2129 °C, and 2227 °C for bioreactors K1, K2, K3, and K4, respectively. The run K3 was the reference for these experiments, thus the accumulated temperatures were 20%, 11%, and 5% higher in bioreactors K1, K2, K4. As accumulated temperature is a time-integrated quantity and its value therefore depends on the duration of the monitoring period, it is not, strictly speaking, an intensive thermodynamic property, and comparisons based on it should be interpreted with caution. To provide a measure that is independent of the observation period and directly comparable between runs, the average temperature recorded over the whole process was also calculated for each bioreactor: 46.2 °C, 44.1 °C, 39.3 °C, and 40.4 °C for K1, K2, K3, and K4, respectively (Figure 1). Relative to the reference run K3, the average temperatures were 18%, 12%, and 3% higher in bioreactors K1, K2, and K4, in line with the trend already observed for the accumulated values.

By day 14 the temperature was found to decrease dramatically in all of the bioreactors and, therefore, the substrate was turned. Turning leads to aeration, homogenization, and improves the structure of the composting substrate. Following this operation, the temperature was observed to increase in all the tested substrates. The resulting new maxima were similar to those noted in the previous phase of the experiment, with the highest temperature of 70 °C in variant K1.

Aeration was controlled individually in each bioreactor with its rate adjusted in order to maximize the temperature. Moreover, increased airflow values resulted in larger amounts of evaporated water. The time course of aeration for each bioreactor is presented in Figure 1. Simultaneous analysis of Figure 1 gives proof that the adopted approach was correct—in order to reach the maximum temperature, the aeration was decreased. This is clearly visible at the beginning of the experiment and in the periods directly preceding and following the turning. When the temperature was found to decrease, the air flowrate was increased.

### 3.2. Mass Reduction

The starting mass was 22 kg in all of the bioreactors. As the biodrying process advanced, the mass of the substrate was found to decrease. Table 3 presents the results of fresh matter, dry matter, and dry organic matter at selected timepoints of the process.

According to the data, the highest loss of substrate mass occurred in bioreactor K2. The mixture subjected to biodrying in this vessel was composed of green waste, vegetables (cabbage/lettuce leaves), kitchen waste, and packaging materials separated from food waste. Losses of 49.09%, 40.94%, 44.01% of fresh matter, dry matter, dry organic matter, respectively, were recorded in this case. Bioreactor K1 showed the second highest loss values. The substrate used in this case consisted of green waste, vegetables (cabbage/lettuce leaves), and packaging materials. The control substrate, K3, comprised of green waste and vegetables (cabbage/lettuce leaves), showed the lowest loss values of 36.36%, 23.56%, and 23.43%, respectively for fresh matter, dry matter, and dry organic matter. Based on these results, it was found that the addition of packaging materials to the substrate increased the mass loss of the treated waste. In a report by Rada et al. (2007) [54] a similar observation was made. Furthermore, for substrates with moisture higher than 40%, mass losses greater than 25% were observed [55]. The reduction of mass is the effect of water evaporation and, to a lesser extent, of the decomposition of organic matter. Higher losses of mass result in obtaining fuel with a better energetic value [56,57]. Of note, the results obtained in real installations may be better. While the construction of the bioreactor used enabled even distribution of air throughout almost the whole substrate, local obstructions existed in the vicinity of the vessel walls. This was manifested in the dry matter measurements. When the homogenized material from the whole vessel was analyzed, the values were 49.91%, 46.26%, 47.44%, 49.57% for K1, K2, K3, K4, respectively. However, when only the fraction from the center of the substrate was weighed the results amounted to 77.65%, 67.02%, 69.76%, 69.85%, respectively. These results indicate that it is possible to obtain dry matter values almost as high as 78%. In another study, a rotary drum system for biodrying was tested [58]. The authors reported mass losses as high as 81%, which, when compared to the results obtained in our study for the homogenized substrate, results in a value almost twice as high.

The masses of collected leachates and condensates should be taken into account when interpreting the mass loss results. The largest mass of all these liquids was collected from bioreactor K2 (6.9 kg), the same one for which the highest mass loss was observed. In the other bioreactors, the effluent masses also corroborated with mass losses of the substrate. Thus, the order of the runs based on the mass of effluents—K2 (6.9 kg), K1 (6.09 kg), K4 (5.74 kg), K3 (4.49 kg)—reflected the one based on mass loss, with bioreactor K2 showing the highest mass reduction and the largest amount of collected effluents, and K3 showing the opposite. Noteworthy to report, in bioreactor K2 no leachate was collected.

### 3.3. Gas Emissions

**Figure 2:**
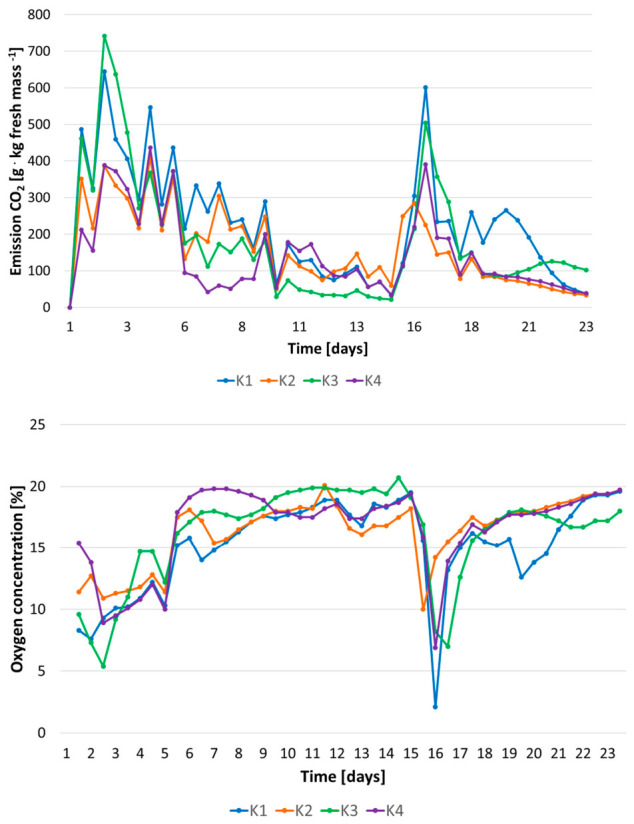
Oxygen and carbon dioxide in the bioreactor off-gases.

The qualitative and quantitative compositions of the gas released during biodrying are important indicators of its proper progress [59]. During the experiment, the contents of oxygen and carbon dioxide in the off-gas were found to be variable (Figure 2). This was a result of both the changes in the flowrate of the supplied air and the biological activity of the microorganisms. The amounts of both these gases are interrelated. In a study similar to ours, oxygen levels exceeding 15% were also reported [55]. The relation between oxygen supply and carbon dioxide production has a deciding effect on the process of composting. The low airflow at the initiation of the experiment, aimed at maximizing the substrate temperature, was reflected in the variability of the oxygen and carbon dioxide contents in the gas exiting the vessel. As a result of this approach, the level of oxygen was low and that of carbon dioxide was high during this stage of the process. When the airflow was kept constant, e.g., between days 5 and 12, the contents of these gases underwent a change. This can be attributed to decreased oxygen demand—the effect of prior biomass decomposition. This was found to manifest in increased oxygen and decreased carbon dioxide levels.

### 3.4. Analysis of the Product—Moisture

**Figure 3:**
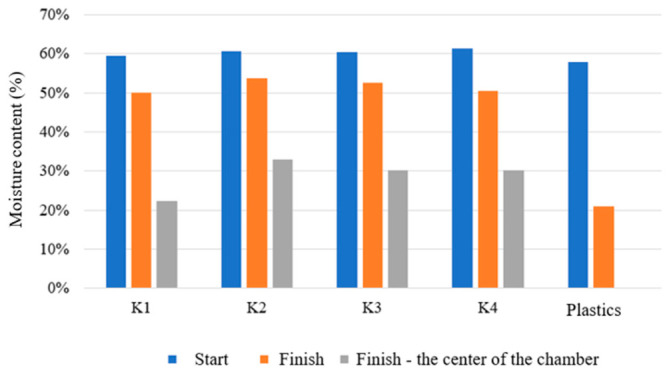
Moisture content in the materials subjected to biodrying, including the results for samples collected from the center of the vessel.

The biodrying process had an effect on the quality of the product—it resulted in a changed moisture content and calorific value. As presented in Figure 3, a loss of moisture was observed in each of the substrates. This is beneficial because, as also shown in other reports, it leads to a simultaneous decrease of the mass of the fuel and an increase of its calorific value [60,61,62].

As mentioned above, the most pronounced decrease in moisture content was observed in the materials located in the center of the vessels. Nonetheless, plastics separated from the mixtures showed a very high loss of moisture of approximately 40% irrespective of the sampling location. This was a result not only of the generally decreased moisture in the substrate but also of the low water absorption capacity of plastics. Moreover, the decomposition of the biodegradable matter initially present in this fraction of the mixtures contributed to this effect. Based on these results, it can be claimed that the addition of plastics to the composting mixes did not impact the biodrying process negatively.

### 3.5. Analysis of the Product—Calorific Value

**Figure 4:**
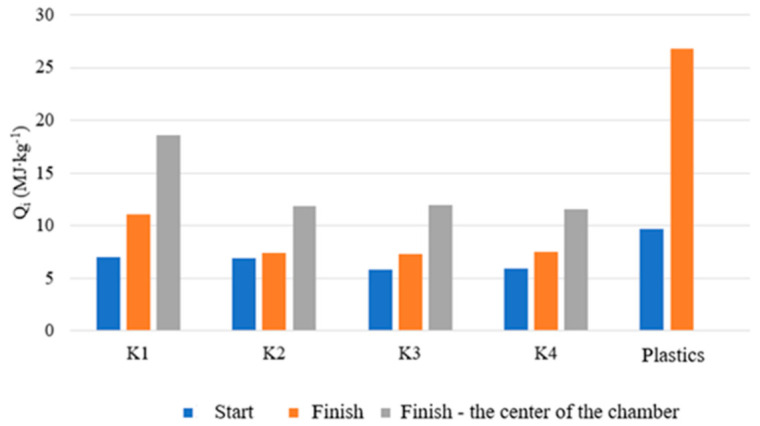
Calorific value of materials subjected to biodrying, including the results for samples collected from the center of the vessel.

Calorific value is the energy released during the combustion of a specified mass of a fuel. It is a parameter directly correlated with the moisture content of the fuel [63,64]. Also in our research, this proved to be true. Comparison of Figure 3 and Figure 4 clearly demonstrates that the decreased moisture in the compost mixes or the plastics translated into increased calorific value.

In the study, the calorific value was determined based on the actual moisture content of the examined materials. The most pronounced difference between the initial and final calorific values was noted for substrate K1. This was true for both the sample from the center of the bioreactor and the homogenized sample. This substrate was characterized by one of the largest moisture losses. It also contained plastics contributing to its high calorific value. The calorific value was found to increase as a result of the biodrying in all the other variants. This is in accordance with the literature as most of the available results show increased calorific values for substrates subjected to biodrying [11,65,66].

It seems worth mentioning that the plastics separated from the biodried material showed the highest increase in calorific value, which exceeded 15 MJ∙kg^−1^. Such an increase of the calorific value was a result of moisture reduction and the decomposition of kitchen waste composed of organic matter. The final calorific value of sample K1 collected from the center of the bioreactor was 18.5 MJ∙kg^−1^ while that of the plastics fraction was 26.8 MJ∙kg^−1^. Thus, it is justified to compare the determined values with other fuels used industrially—wood, bituminous coal, pellet, or RDF. Alternative fuels, such as RDF, typically have calorific values of 18 MJ∙kg^−1^ [67,68]. Furthermore, the result obtained for the K3 sample makes it comparable to wood or pellet—the calorific values of which are 17 and 18.8 MJ∙kg^−1^, respectively [69,70]. Resources such as bituminous coal with a calorific value of 32 MJ∙kg^−1^ may become replaced by plastics, but for this to happen, suitable technologies are required [69,71].

The biodrying process did not influence the elementary composition of the materials significantly (Table 4). The ratios of hydrogen to carbon, and of oxygen to carbon are typical for biomass that has not been subjected to thermal treatment. The ratio of carbon to nitrogen is another noteworthy parameter that is characteristic of composting [72].

### 3.6. Analysis of the Product—Compost Quality

**Figure 5:**
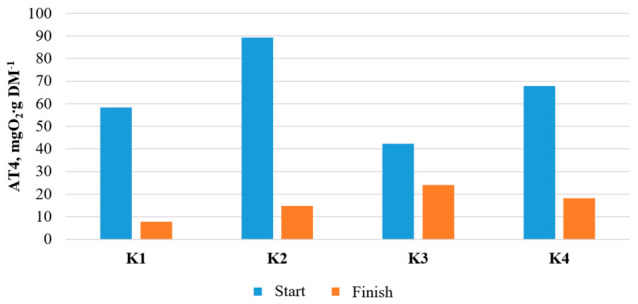
AT4 values of the tested variants with respect to dry matter.

To confirm the expected high degree of decomposition of the biodegradable fractions of the substrate, AT4 tests were performed in samples collected before the process of biodrying and after it. The results are presented in the following Figure 5. Based on the results of these tests it can be stated that the biodrying process resulted in significant biodegradation of the biologically reactive constituents as the values of AT4 were greatly reduced following this treatment. In the samples obtained from bioreactors K1 and K2, the final AT4 values were close to 10 mg O_2_∙g DM^−1^. In another study, such a value, indicating stability of the product, was obtained after 90 days [48]. This level of the AT4 parameter reflects a low potential of the compost to further decompose and makes it suitable for transport and prolonged storage without the emission of odors. Furthermore, the comparison of the results obtained for K1 and K2 with the control runs demonstrates that the addition of plastics to biodegradable biomass did not impact the composting process negatively. Instead, a positive effect was observed—the values of AT4 reduction were 87% and 83% in K1 and K2, respectively, while those observed for K3 and K4 were 43% and 73%, respectively. It should be noted, however, that AT4 thresholds used to define a “stable” or mature material for soil-improver applications typically range from 5 to 10 mg O_2_ g^−1^ DM, depending on the regulatory framework considered. On this basis, the K1 and K2 products (AT4 close to 10 mg O_2_ g^−1^ DM) approach the upper end of this stability range, whereas K3 and K4 remain further from it. This indicates that, under the tested conditions, the plastic-amended variants moved closer to meeting soil-improver maturity criteria than the controls; although a formal comparison against a specific national or EU soil-improver standard was beyond the scope of this study, it is recommended for future work. It must also be stressed that each bioreactor variant (K1–K4) was run without process replication; therefore, the AT4, temperature, and mass loss differences reported between variants should be regarded as indicative trends from a single experimental run rather than statistically validated effects and should be interpreted with appropriate caution.

### 3.7. Discussion

The higher cumulative temperature recorded for K1 compared with the K3 control can plausibly be attributed to two combined factors. First, the presence of plastic fragments increased the bulk density and improved the structure of the substrate mixture, which is likely to have facilitated a more even airflow distribution and a reduced short-circuiting of aeration through the bed, allowing more heat to be retained rather than being removed by the air stream. Second, exothermic oxidative degradation of the plastic surfaces themselves, in addition to microbial oxidation of the biodegradable fraction, may have contributed an additional heat source. The relative contribution of these two mechanisms (aeration-related heat retention versus additional oxidative heat release) could not be separated within the scope of the present single-run experiments, and their disentanglement is recommended as a direction for future, replicated studies.

In addition to the main temperature peak observed during the first 48 h and the secondary peak following the turning on day 14, smaller temperature rebounds were observed around day 7 and again around days 18–20 in several of the bioreactors. These secondary rises are consistent with the activity of slower-growing microbial populations becoming dominant once the readily degradable fraction of the substrate has been depleted, as well as with localized re-aggregation of moisture and substrate within the bed creating new pockets favorable to microbial activity. While these rebounds were of smaller magnitude than the main peaks and did not materially change the overall mass loss or AT4 outcomes, they illustrate that the biodrying process did not proceed as a single monotonic decay and that intermediate turning or aeration adjustments could be used in future work to manage these secondary activity phases.

In this laboratory-scale experiment, the plastic fraction was separated from the biodried material manually on a sieve, which proved labor-intensive. At an industrial scale, manual sorting would not be economically viable, and separation would need to rely on established mechanical sorting technologies already used in waste-to-fuel processing lines, such as near-infrared (NIR) optical sorters, which distinguish plastics from organic material based on spectral reflectance, and density-based separators (e.g., air classifiers or ballistic separators); the latter exploit the lower density and different aerodynamic behavior of dried plastic fragments relative to the biodegradable matrix. Integration of such technologies would be a necessary step before the approach described here could be scaled beyond the laboratory and represents an important direction for future techno–economic assessment.

It should also be noted that, although biodrying relies on biological heat generation rather than external energy input, thermal drying processes used elsewhere in waste valorization chains are energy-intensive and can carry substantial environmental burdens. A recent prospective life-cycle assessment of waste drying for biorefinery purposes found that the climate-change impact of drying could vary from 0.42 to 0.11 kg CO_2_-eq per kg of evaporated water depending on the energy carrier and process temperature used, with capital goods contributing up to 36.9% of this impact at higher drying temperatures [73]. This underlines the environmental advantage of biodrying, which avoids dedicated drying energy inputs altogether, and suggests that a full life-cycle assessment comparing biodrying with conventional thermal drying of the plastic-containing fraction would be a valuable direction for future work.

## 4. Conclusions

The results obtained for the biodrying process carried out prove that the experiment was conducted correctly. This was confirmed by the parameters related to the change in temperature and the differences between the concentrations of oxygen and carbon dioxide emitted during the process, as well as the mass loss in each of the investigated mixtures. In addition, the use of the biodrying process had a positive effect on improving the calorific value of plastics, which can be a suitable material for the production of RDF fuels. The main reason for the increase was a significant reduction in moisture content from 58% to 21%. This factor improved the calorific value from 9.6 to 26.8 MJ∙kg^−1^. However, it should be noted that there were difficulties in separating the plastics on the sieve. This concerned the relatively small distribution of dry hay contained in the green waste, which was characterized by a similar granulometric size; therefore, the plastic separation process was done manually.

The plastics had a bulk density of 150 kg·m^−3^, which contributed to the bulk density of the individual feedstocks. This was certainly due to the nature of the plastics, to which the dry grass material stuck, increasing its bulk density. Moreover, the addition of plastics contributed to a higher maximum feedstock temperature (72.94 and 70.4 °C) compared to samples without their addition (70.19 and 69.56 °C). It also contributed to 11% and 20% higher cumulative temperatures (corresponding to 12% and 18% higher average temperatures, respectively) in the cases with the addition of plastics—a difference that, as discussed in Section 3.7, is likely attributable to a combination of improved aeration-related heat retention and additional oxidative heat release from the plastic fraction. Furthermore the samples with the addition of plastics were characterized by a significantly higher reduction in dry organic matter. These preliminary, single-run results suggest that pre-treated plastics recovered from the mechanical unpacking of food waste may have potential as a structuring material in biodrying systems, as an alternative to wood chips. This finding applies specifically to the biodrying context studied here, where the end product is intended as a solid fuel, and should not be extended to agricultural composting aimed at producing organic fertilizers or soil improvers, where the presence of plastic residues in the final product would be undesirable. Confirmation in replicated trials, together with an assessment of any associated microplastic release, would be required before this application could be recommended more broadly.

## Figures and Tables

**Table 1:** Initial physicochemical characteristics of substrates (own elaboration).

Substrates	pH	Dry Matter (%)	Dry Organic Matter (% DM)	Bulk Density (kg·m^−3^)
Green biomass	6.35	71.87	91.12	90
Vegetables (cabbage/lettuce leaves)	5.77	7.12	81.81	320
Kitchen waste	4.09	31.93	93.92	1040
Packaging materials separated from food waste	5.26	42.53	95.88	150

**Table 2:** Initial parameters of substrates (own elaboration).

Parameter	Designation			
	K1	K2	K3 (Control)	K4
Composition	- Green biomass - Vegetables (cabbage/lettuce leaves) - Packaging materials separated from food waste	- Green biomass - Vegetables (cabbage/lettuce leaves) - Kitchen waste - Packaging materials separated from food waste	- Green biomass - Vegetables (cabbage/lettuce leaves)	- Green biomass - Vegetables (cabbage/lettuce leaves) - Kitchen waste
Dry matter percentage of the fractions (%)	62/6/32	57/6/6/31	91/9	84/8/8
Raw mass of the fractions (kg)	7.7/7.7/6.6	6.93/6.93/1.54/6.6	11/11	9.9/9.9/2.2
Dry matter (%)	40.4	39.9	39.5	38.7
Moisture (%)	59.6	60.13	60.51	61.26
Bulk density (kg·m^−3^)	279	294	241	259
Volume (dm^3^)	79	74.9	91.1	85.1

**Table 3:** Mass changes during the biodrying process (own elaboration).

**Fresh Matter (kg)**				
	**K1**	**K2**	**K3**	**K4**
Start	22.00	22.00	22.00	22.00
Turning	15.55	14.15	16.90	15.85
Finish	12.55	11.20	14.00	12.80
Loss (%)	42.95	49.09	36.36	41.82
**Dry Matter (kg)**				
	**K1**	**K2**	**K3**	**K4**
Start	8.89	8.77	8.69	8.52
Turning	9.03	7.92	10.02	8.62
Finish	6.26	5.18	6.64	6.35
Loss (%)	29.53	40.94	23.56	25.54
**Dry Organic Matter (kg)**				
	**K1**	**K2**	**K3**	**K4**
Start	8.18	8.09	7.84	7.72
Turning	8.20	6.91	8.95	7.67
Finish	5.59	4.53	6.01	5.71
Loss (%)	31.71	44.01	23.43	26.00

**Table 4:** Percentage content of selected elements, C/N ratio, and molar ratios of H/C and O/C in samples before and after biodrying process.

	C (%)	TOC (%)	H (%)	N (%)	S (%)	O (%)	H/C	O/C	C/N
K1—start	46	46	6	3	0.2	34	1.65	0.55	15.49
K2—start	47	47	6	3	0.2	34	1.67	0.55	14.80
K3—start	44	44	6	2	0.2	35	1.57	0.59	28.58
K4—start	45	44	6	2	0.2	35	1.59	0.59	25.36
K1—finish	49	47	7	2	0.0	31	1.64	0.47	26.81
K2—finish	45	45	6	3	0.3	33	1.65	0.55	16.01
K3—finish	45	41	6	2	0.2	38	1.50	0.64	23.90
K4—finish	44	42	6	2	0.2	39	1.54	0.67	24.07

## Data Availability

The original contributions presented in this study are included in the article. Further inquiries can be directed to the corresponding author.

## Abbreviations

The following abbreviations are used in this manuscript

AT4	total oxygen consumption after 4 days
DM	dry matter
RDF	refuse derived fuel
SRF	solid recovered fuels

## References

[1] Tajima T., Necas A., Massard T., Gales S. (2022). East Meets West Again in Order to Tackle the Global Energy Crisis. Uspekhi Fiz. Nauk.

[2] Turiel A. (2022). The Energy Crisis in the World Today: Analysis of the World Energy Outlook. https://doi.org/10.31009/viewpoint.2022.01.

[3] Selvanathan A., Vijayaragavan M. (2023). Effect on Minor Addition of Aromatic (Benzyl Alcohol) and Diethyl Ether in Calophyllum inophyllum Blended Diesel Fuel in a CI Engine Operates by Hydrogen Energy as a Secondary Fuel. Energy.

[4] Czekała W. (2021). Solid Fraction of Digestate from Biogas Plant as a Material for Pellets Production. Energies.

[5] Singh S. (2021). Energy Crisis and Climate Change: Global Concerns and Their Solutions. Energy Crises: Challenges and Solutions.

[6] Poudyal R., Loskot P., Nepal R., Parajuli R., Khadka S.K. (2019). Mitigating the Current Energy Crisis in Nepal with Renewable Energy Sources. Renew. Sustain. Energy Rev..

[7] Daggash H.A. (2022). How the Global Energy Crisis Is Improving the Prospects of Solar in Nigeria. https://energyforgrowth.org/article/how-the-global-energy-crisis-is-improving-the-prospects-of-solar-in-nigeria/.

[8] UNCTAD (2026). Strait of Hormuz Disruptions: Implications for Global Trade and Development.

[9] Farghali M., Osman A.I., Mohamed I.M.A., Chen Z., Chen L., Ihara I., Yap P.S., Rooney D.W. (2023). Strategies to save energy in the context of the energy crisis: A review. Environ. Chem. Lett..

[10] Liang Z., Yu Z., Liu H., Chen L., Huang X. (2022). Combustion and Emission Characteristics of a Compression Ignition Engine Burning a Wide Range of Conventional Hydrocarbon and Alternative Fuels. Energy.

[11] Li Y., Ma J., Yong X., Luo L., Wong J.W.C., Zhang Y., Wu H., Zhou J. (2022). Effect of Biochar Combined with a Biotrickling Filter on Deodorization, Nitrogen Retention, and Microbial Community Succession during Chicken Manure Composting. Bioresour. Technol..

[12] van Ruijven B.J., De Cian E., Sue Wing I. (2019). Amplification of Future Energy Demand Growth Due to Climate Change. Nat. Commun..

[13] Witaszek K., Herkowiak M., Pilarska A.A., Czekała W. (2022). Methods of Handling the Cup Plant (*Silphium perfoliatum* L.) for Energy Production. Energies.

[14] Hou R., Deng G., Wu M., Wang W., Gao W., Chen K., Liu L., Dehan S. (2023). Optimum Exploitation of an Integrated Energy System Considering Renewable Sources and Power-Heat System and Energy Storage. Energy.

[15] Song X., Zhang H., Fan L., Zhang Z., Peña-Mora F. (2023). Planning Shared Energy Storage Systems for the Spatio-Temporal Coordination of Multi-Site Renewable Energy Sources on the Power Generation Side. Energy.

[16] Frankowski J., Czekała W. (2023). Agricultural Plant Residues as Potential Co-Substrates for Biogas Production. Energies.

[17] Parsaee M., Kiani M.K.D., Karimi K. (2019). A Review of Biogas Production from Sugarcane Vinasse. Biomass Bioenergy.

[18] Czekała W. (2023). Selective Collection and Management of Biowaste from the Municipal Sector in Poland: A Review. Appl. Sci..

[19] Nwokolo N., Mukumba P., Obileke K., Enebe M. (2020). Waste to Energy: A Focus on the Impact of Substrate Type in Biogas Production. Processes.

[20] Dach J., Pulka J., Janczak D., Lewicki A., Pochwatka P., Oniszczuk T. (2020). Energetic Assessment of Biogas Plant Projects Based on Biowaste and Maize Silage Usage. IOP Conf. Ser. Earth Environ. Sci..

[21] Ferdeș M., Dincă M.N., Moiceanu G., Zăbavă B.Ș., Paraschiv G. (2020). Microorganisms and Enzymes Used in the Biological Pretreatment of the Substrate to Enhance Biogas Production: A Review. Sustainability.

[22] Sun Y., Yang B., Wang Y., Zheng Z., Wang J., Yue Y., Mu W., Xu G., Ying J. (2023). Emergy Evaluation of Biogas Production System in China from Perspective of Collection Radius. Energy.

[23] Scherhaufer S., Moates G., Hartikainen H., Waldron K., Obersteiner G. (2018). Environmental Impacts of Food Waste in Europe. Waste Manag..

[24] Borowski S., Boniecki P., Kubacki P., Czyżowska A. (2018). Food Waste Co-Digestion with Slaughterhouse Waste and Sewage Sludge: Digestate Conditioning and Supernatant Quality. Waste Manag..

[25] Garcia N.H., Mattioli A., Gil A., Frison N., Battista F., Bolzonella D. (2019). Evaluation of the Methane Potential of Different Agricultural and Food Processing Substrates for Improved Biogas Production in Rural Areas. Renew. Sustain. Energy Rev..

[26] Marks S., Dach J., Morales F.J.F., Mazurkiewicz J., Pochwatka P., Gierz Ł. (2020). New Trends in Substrates and Biogas Systems in Poland. J. Ecol. Eng..

[27] (1923). Rozporządzenie Ministra Środowiska z dnia 9 grudnia 2014 r. w sprawie katalogu odpadów. Dz. U. 2014, poz. https://isap.sejm.gov.pl/isap.nsf/DocDetails.xsp?id=wdu20140001923.

[28] Szewski A., Budziszewski A. (2023). Ewidencja odpadów 2023. Poradnik BDO. https://kartaewidencji.pl/ewidencja-odpadow-2020-poradnik-przedsiebiorcy/.

[29] Scarlat N., Fahl F., Dallemand J.F. (2019). Status and Opportunities for Energy Recovery from Municipal Solid Waste in Europe. Waste Biomass Valorization.

[30] Jang Y.C., Lee G., Kwon Y., Lim J.H., Jeong J.H. (2020). Recycling and Management Practices of Plastic Packaging Waste towards a Circular Economy in South Korea. Resour. Conserv. Recycl..

[31] Kumar A., Samadder S.R. (2023). Development of Lower Heating Value Prediction Models and Estimation of Energy Recovery Potential of Municipal Solid Waste and RDF Incineration. Energy.

[32] Bojarski W., Pulka J., Pochwatka P., Bresz P., Nowak M., Dach J. (2023). Energetic Potential of Dairy Cow Breeding in Poland. Farm Machinery and Processes Management in Sustainable Agriculture.

[33] Xin L., Qin Y., Lou T., Xu X., Wang H., Mei Q., Wu W. (2023). Rapid Start-Up and Humification of Kitchen Waste Composting by an Innovative Biodrying-Enhanced Process. Chem. Eng. J..

[34] Yu B., Chen T., Wang X., Yang J., Zheng G., Fu L., Huang X., Wang Y. (2023). Biodrying Characteristics and Microbial Community Succession. Sci. Total Environ..

[35] Zhang G., Guo X., Zhu Y., Liu X., Han Z., Sun K., Ji L., He Q., Han L. (2018). Effects of Different Biochars on Microbial Quantity and Biodegradation. Geoderma.

[36] Tun M.M., Juchelková D. (2019). Drying methods for municipal solid waste quality improvement in the developed and developing countries: A review. Environ. Eng. Res..

[37] Dos Reis R.F., Cordeiro J.S., Font X., Laguna Achon C. (2020). The biodrying process of sewage sludge—A review. Dry. Technol..

[38] Rolfe A., Huang Y., Hewitt N. Technical and Environmental Analysis of Methanol Synthesis from Solid Recovered Fuel and Lignite. Proceedings of the 8th International Conference on Sustainable Solid Waste Management.

[39] Shehata N., Obaideen K., Sayed E.T., Abdelkareem M.A., Mahmoud M.S., El-Salamony A.H.R., Mahmoud H.M., Olabi A.G. (2022). Role of refuse-derived fuel in circular economy and sustainable development goals. Process Saf. Environ. Prot..

[40] Haar Q. (2023). Refuse Derived Fuels (RDF) and Solid Recovered Fuels (SRF): A Case Study of Characteristics and Opportunities. Master’s Thesis.

[41] Czekała W., Janczak D., Pochwatka P., Nowak M., Dach J. (2022). Gases emissions during composting process of agri-food industry waste. Appl. Sci..

[42] Alkoaik F.N., Abdel-Ghany A.M., Rashwan M.A., Fulleros R.B., Ibrahim M.N. (2018). Energy analysis of a rotary drum bioreactor for composting tomato plant residues. Energies.

[43] Bajko J., Fišer J., Jícha M. (2018). Temperature measurement and performance assessment of the experimental composting bioreactor. EPJ Web Conf..

[44] Junne S., Neubauer P. (2018). How scalable and suitable are single-use bioreactors?. Curr. Opin. Biotechnol..

[45] Xin L., Yan X., Xu X., Qin Y., Nan Q., Wang H., Wu W. (2022). Carbohydrate degradation contributes to the main bioheat generation during kitchen waste biodrying process: A pilot study. Waste Manag..

[46] (2019). Sludge, Treated Biowaste, Soil and Waste—Determination of Loss on Ignition.

[47] (2017). General Requirements for the Competence of Testing and Calibration Laboratories.

[48] (2017). Solid Biofuels—Determination of Calorific Value.

[49] Akyol Ç., Ince O., Ince B. (2019). Crop-based composting of lignocellulosic digestates: Focus on bacterial and fungal diversity. Bioresour. Technol..

[50] Meng X., Yan J., Zuo B., Wang Y., Yuan X., Cui Z. (2020). Full-scale of composting process of biogas residues from corn stover anaerobic digestion. Bioresour. Technol..

[51] Zhao Y., Li W., Chen L., Meng L., Zhang S. (2023). Impacts of adding thermotolerant nitrifying bacteria on nitrogenous gas emissions and bacterial community structure during sewage sludge composting. Bioresour. Technol..

[52] Droffner M.L., Brinton W.F.J., Evans E. (1995). Evidence for the prominence of well characterized mesophilic bacteria in thermophilic composting environments. Biomass Bioenergy.

[53] Wang Y., Akdeniz N. (2024). Mathematical modeling of biochar’s role in elevating co-composted poultry carcass temperatures. Waste Manag..

[54] Rada E.C., Franzinelli A., Taiss M., Ragazzi M., Panaitescu V., Apostol T. (2007). Lower heating value dynamics during municipal solid waste bio-drying. Environ. Technol..

[55] Vigano F., Consonni S., Ragazzi M., Rada E.C. A model for mass and energy balances of bio-drying. Proceedings of the North American Waste-to-Energy Conference.

[56] Silva J.D.O., Santos D.E.L., Souza Abud A.K., Oliveira Júnior A.M. (2020). Characterization of acerola industrial waste as raw material for thermochemical processes. Waste Manag..

[57] Feltrim F., Izzo R.L.S., Rose J.L., Machado A.B., Oro S.R. (2021). Evaluation of the bio-drying process of municipal solid waste using rotating drums. An. Acad. Bras. Ciênc..

[58] González D., Guerra N., Colón J., Gabriel D., Ponsá S., Sánchez A. (2019). Filling in sewage sludge biodrying gaps: Greenhouse gases, VOCs and odour emissions. Bioresour. Technol..

[59] Oh K.C., Kim J., Park S.Y., Kim S.J., Cho L.H., Lee C.G., Roh J., Kim D.H. (2021). Development and validation of torrefaction optimization model. Energy.

[60] Yansen A. (2021). Alternative industrial fuels in Indonesia from urban waste treatment using RDF through bio-drying. Proceedings of the International Conference on Science and Engineering (ICSE-UIN-SUKA 2021).

[61] Kijo-Kleczkowska A., Szumera M., Gnatowski A., Sadkowski D. (2022). Comparative thermal analysis of coal fuels, biomass, fly ash and polyamide. Energy.

[62] Tumuluru J.S., Yancey N.A., Kane J.J. (2021). Pilot-scale grinding and briquetting studies on MSW. Waste Manag..

[63] Turzyński T., Januszewicz K., Kazimierski P., Kardaś D., Hercel P., Szymborski J., Niewiadomski J. (2023). Role of additives in improving flammability and calorific value. Waste Manag..

[64] Yuan J., Li Y., Wang G., Zhang D., Shen Y., Ma R., Li D., Li S., Li G. (2019). Biodrying performance and combustion characteristics. Bioresour. Technol..

[65] Xin L., Li X., Bi F., Yan X., Wang H., Wu W. (2021). Accelerating food waste composting with biodrying. ACS Sustain. Chem. Eng..

[66] Zhao L., Giannis A., Lam W.Y., Lin S.X., Yin K., Yuan G.A., Wang J.Y. (2016). Characterization of Singapore RDF resources. Sustain. Environ. Res..

[67] Smoliński A., Wojtacha-Rychter K., Król M., Magdziarczyk M., Polański J., Howaniec N. (2022). Co-gasification of RDF and coal. Energy.

[68] Hamzah N., Zandi M., Tokimatsu K., Yoshikawa K. (2017). Woody biomass characterization. Energy Procedia.

[69] Asibor J.O., Akhator E.P., Obanor A.I. (2019). Energy potential study of tropical wood species. Curr. Appl. Sci. Technol..

[70] Muthukumar K., Kasiraman G. (2023). Utilization of fuel energy from plastic waste. Energy.

[71] Cáceres R., Malińska K., Marfà O. (2018). Nitrification within composting: A review. Waste Manag..

[72] Sánchez Arias V., Fernández F.J., Rodríguez L., Villaseñor J. (2012). Respiration indices and stability measurements of compost. J. Environ. Manag..

[73] Niedzwiecki L., Kupka D., Cespiva J., Skrinsky J., Fiori L. (2026). Prospective LCA-based optimisation of tomato waste drying for biorefinery purposes. Appl. Energy Combust. Sci..

